# Evaluating hepatocellular carcinoma (HCC) surveillance through an early diagnostic centre: An implementation science approach at a tertiary hepatology centre in England

**DOI:** 10.1016/j.clinme.2025.100531

**Published:** 2025-11-13

**Authors:** Hamzah Z. Farooq, Kevin Galono, Leila Reid, Janet Dearden, Graham R. Foster

**Affiliations:** a Blizard Institute, Queen Mary University of London, London, UK; b Department of Hepatology, Barts Health NHS Trust, London, UK; c Department of Infectious Diseases, Manchester University NHS Foundation, Manchester, UK; d Department of Virology, UKHSA Manchester, Manchester, UK; e The Hepatitis C Trust, London, UK

**Keywords:** Hepatocellular carcinoma, Surveillance, Cancer diagnosis

## Abstract

**Background:**

Hepatocellular carcinoma (HCC) has high mortality, improved by early diagnosis. An integrated service was established to facilitate timely cancer surveillance*.*

**Aims:**

Evaluate the utility of an early diagnostic centre (EDC) in diagnosis of liver cancer.

**Methods:**

Retrospective analysis of patients at an EDC (2022–2024), compared to previous general clinic attendance. We investigated demographics, and liver disease aetiology and status. We tracked attendances and ‘did-not-attend’ rates, with sub-categorisation by time period. Assessments were categorised as ideal, optimal, sub-optimal or poor. Travel-costs and times were estimated.

**Results:**

Patients attending the centre (315) were reviewed, with previous clinic reviews serving as comparator. The cohort was predominantly male (58.1%), mean age 53.93 ± 11.75 years, British-African descent (20.3%), from deprived backgrounds (68.8%) and non-cirrhotic (53.3%). The leading liver disease aetiology was hepatitis B (54.3%), followed by hepatitis C (20.6%) and alcoholic liver disease (11.4%).

The EDC had a lower ‘did-not-attend’ rate (15.70% vs. 23.97% in general clinics), higher proportion of ideal assessments (99.7% vs. 51.1%), lower ‘did-not-attend’ costs (£11,695 vs. £119,796) and lower patient travel time (461 vs. 655 h) and cost (£3,249 vs. £9,765). Sub-stratification by COVID time periods showed 55 attendance failures pre-COVID, 146 during COVID and 401 post-COVID; compared to 73 ‘did-not-attends’ in the EDC.

**Conclusion:**

The EDC demonstrated clinical and economic efficiencies, improved surveillance and a reduction in costs compared to traditional clinics. These results underscore the potential of this model to enhance HCC surveillance, reduce costs and improve compliance.

## Highlights

• Hepatocellular carcinoma (HCC) is associated with high global morbidity and mortality.
• A dedicated integrated HCC surveillance clinic was implemented at a tertiary hepatology centre.
• Clinical and economic efficiencies, improved HCC surveillance and a reduction in costs occurred in this model, compared to traditional clinics.
• The integrated cancer screening model shows strong potential to improve cancer screening uptake, optimise surveillance, and lower overall healthcare costs.
• Establishing a ‘gold standard’ for HCC surveillance is crucial for ensuring effective reviews of people at risk of HCC.

## Introduction

Hepatocellular carcinoma (HCC) is a major global cause of morbidity and mortality. With the global obesity pandemic, rising prevalence of metabolic-associated steatotic liver disease (MASLD) and ongoing hepatitis B virus (HBV) transmission in marginalised populations, HCC incidence is projected to rise by 55%, with 1.4 million cases and 1.3 million deaths by 2040.^1^

As the 5-year survival rate of early-stage HCC is 75% compared to 5% in late-stage,^2^, ^3^, ^4^ early diagnosis is important for treatment,^5^ as recommended by the independent NHS review panel NICE (National Institute of Health and Care Excellence). The American,^6^ British^7^ and European^2^ liver societies have issued guidelines recommending HCC surveillance every 6 months, with patients reviewed by a healthcare professional (HCP) with imaging (ultrasonography) and blood tests including alpha-fetoprotein (AFP) to ensure that early HCC lesions are detected and treated. Poor surveillance uptake may be influenced by patient, clinician or systemic factors.^8^ Liver disease disproportionately affects those from lower socio-economic groups with associated low health literacy^9^ and the best way to achieve high rates of effective surveillance in people with liver disease is unclear.

The hepatology department in the Royal London Hospital (RLH) at Barts Health NHS Trust is a tertiary hepatology centre serving a deprived community in East London, which has traditionally monitored for HCC in general hepatology outpatient clinics for patients from across London.

To ensure timely surveillance and improve HCC prognosis, we established a single destination integrated clinic embedded within an early diagnostic centre (EDC) at an adjacent hospital (Mile End Hospital (MEH)). Equipped with endoscopy, phlebotomy, ultrasound, CT scan and MRI services, it offers patients a convenient single site where they will undergo a blood test, scan and nurse review at the same location. Nurse review can be either face to face or virtual (depending on patient preferences), via telephone with referral to a doctor, if needed.

In this implementation science study, we evaluate the effectiveness and utility of the EDC and its impact on patients attending the service.

## Methods

### EDC model

Patients over 18 years undergoing HCC surveillance as deemed to be at risk and met specific inclusion criteria (Supplementary Appendix 1) were referred to the EDC. Patients were scheduled for a liver USS and blood test on the same day with either a virtual or face-to-face (depending on patient preferences) HCP appointment (specialist hepatology nurse with remote hepatology consultant oversight) 2 weeks after the investigations. If any significant result is noted in the investigations, then the HCP contacts the patient on the same day of the result. On clinical review, if there are no significant findings, the patient will continue with EDC surveillance in 6–12 months’ time. If there are significant findings, then the patient will be escalated to a 2-week cancer pathway.

### Study population

In this single-centre retrospective study, we reviewed all patients who presented to the EDC from its inception (1 November 2022) until data lock on 15 March 2024. For these EDC patients, we retrospectively reviewed their previous attendance in the general hepatology (GH) clinics to compare HCC surveillance before EDC inception. Utilising the electronic health records (EHR) of Barts Health NHS Trust, the data for patients attending EDC were extracted and details (demographics, ethnicity (recorded as per Office for National Statistics criteria) and postcode) were recorded, along with referral route to the EDC, dates of: data capture, referral to the EDC, when the patient was first scheduled/seen in the EDC, and the start of GH HCC surveillance review. As a comparator, we used GH clinic attendance and as the data period of the EDC spanned 17 months, we used an enhanced period prior to the EDC initiation for comparison. Utilising patient postcode data, socioeconomic status was assessed using the Index of Multiple Deprivation (IMD), which ranks small areas in England based on factors including income, employment, education, health, crime, housing, and environment. IMD scores were grouped into (deciles/quintiles), with lower scores indicating higher deprivation

Data collection for GH reviews began from 1 January 2019, or at the first review thereafter, until 15 March 2024. This starting point was to ensure at least 1 year of data collection before the onset of the COVID-19 pandemic in 2020, when a higher proportion of patients were reviewed virtually.​ Patient visits for research studies and those attending for HCV directly-acting-antiviral therapy were excluded.

Data were collected on the primary liver aetiology for EDC surveillance, concurrent liver disease, cirrhosis status, blood parameters at the initial EDC referral (including AFP, bilirubin, ALT, ALP, AST, albumin and platelets) and highest Child–Pugh score. Upon referral to the EDC, the referring clinician indicated whether the patient had cirrhosis, based on either transient elastography findings or histological diagnosis. Due to clinical workload constraints within the NHS, a Child–Pugh score was not recorded for every patient at the time of referral. To address this, we reviewed the clinical notes and available blood results for EDC patients and retrospectively calculated and updated the Child–Pugh scores. Information on HCV treatment and outcome were included if appropriate. The number of attendances in GH pre-EDC (booked/attended), ‘did-not-attends’ (DNAs; total number/face-to-face/virtual appointments) pre-EDC, and the number of hospital or patient cancellations were recorded. Additionally, data on BMI, smoking status and alcohol intake were extracted, with the country of origin ascertained to confirm ethnicity from the initial referral letter and/or appointment.

Dates of the last appointment, blood tests, and scans pre-EDC were noted, along with the dates of all GH appointments pre-EDC until 1 January 2019. The development and staging of HCC (as per the Barcelona Clinic Liver Cancer (BCLC) staging criteria), referral time from HCC onset to treatment, the outcome and status of the patient were documented.

### Statistical analysis

Additional metrics were calculated and captured based on the recorded data. The number of days reviewed in the EDC and GH were calculated, the days from the initial EDC referral to review, the DNA rates at both location and appointment settings (EDC/GH and virtual/face-to-face), and days between each GH review were captured to compare with the recommended review target of 180 days.

From the dates of the last doctor review, blood test and imaging scan before the EDC review, a mid-point was calculated and it was determined whether the scan, appointment and blood test occurred during the recommended HCC surveillance window. The HCC surveillance window was determined as being within 4 weeks for both the scan and blood test and within 6 weeks for the doctor review. From this, we determined whether the patient had an ideal (doctor review, scan and bloods within window), optimal (scan and bloods within window), sub-optimal (doctor review only) or poor (no parameter within window) HCC assessment. This was reviewed with a patient support group representative (L Reid from the Hepatitis C Trust, who have co-produced this paper) to confirm that these windows are in line with patient expectations. Utilising these parameters, we also calculated the days taken for full assessment of HCC surveillance.

The aMAP score (assesses the 5-year HCC risk^10^) was calculated and the score combined with ethnicity classifies individuals as low- (0–49.9), medium- (50–59.9) or high-risk (≥60).^11^

To identify factors associated with an incomplete HCC assessment, (optimal, sub-optimal and poor HCC assessment) compared to a complete HCC assessment (ideal HCC assessment), we first fitted univariate models using a list of covariates. We then adjusted for a predefined subset of covariates, which we considered potential confounders, in a binary logistic regression model to calculate odds ratios (OR) with 95% confidence intervals.

### Patient cost and time to appointment (ToA)

Postcodes were utilised to determine lower layer super output areas (LSOAs), which were applied to find socio-economic data for each patient via ONS data.^12^ To determine crude patient costs and travel time to appointment (ToA), we utilised Google Maps for travel between the patient’s home address (postcode only) to the RLH and MEH locations for peak time (arriving for 0900) cost and time. If these data were not available in Google Maps, we utilised the popular CityMapper app^13^ for travel in London and National Rail for travel outside London. The patient cost and ToA calculations are further detailed in Supplementary Appendix 2.

Utilising the National Cost Collection (NCC) for the NHS,^14^ we calculated the total cost of DNAs for consultant-led appointment, USS and bloods and for the EDC. This was performed by utilising the ‘national average unit cost’ for consultant-led follow-up appointment and for the EDC appointment which is CNS-led – Supplementary Appendix 3.

### DNAs rates secondary to COVID

To review the potential effect of the COVID pandemic and to mitigate a potential subjective comparison between virtual and face-to-face clinic attendances, we captured the number of DNAs for GH clinics during the pre-COVID (face-to-face appointments), COVID (virtual appointments), post-COVID (mixed virtual and face-to-face appointments) periods (defined in Supplementary Appendix 4) with a comparison to the EDC period. Utilising the number of total number of DNAs during these periods, we calculated the rate of DNAs per patient for the pre-COVID, COVID, post-COVID and EDC periods.

Analyses were conducted using R-studio (R Project for Statistical computing), Jamovi (version 2.6.2.0) and Tableau Desktop (version 2024.3.1).

## Results

From 1 November 2022 to 15 March 2024, 328 patients were referred to the EDC. Post review, 12 of these patients were not seen in the EDC as they did not fit the EDC criteria and were excluded resulting in 315 evaluated patients, of whom all opted for virtual clinical review.

### Patient demographics

Of the 315 patients, most (183, 58.1%) were male (Table 1, Supplementary Table 1) with an average age of 53.93 with a high BMI (mean 28.85 ± 7.22) (Supplementary Table 2).

**Table 1:** Demographics of cohort.

Category	Counts	Percent of total	Cumulative percentage
Gender			
Male/female	183/132	58.1%/41.9%	100%
Ethnicity			
Asian – Any other Asian background	25	7.9%	7.9%
Asian or Asian British – Bangladeshi	42	13.3%	21.2%
Asian or Asian British – Pakistani	24	7.6%	28.8%
Black or Black British – African	64	20.3%	49.1%
White – Any other White background	34	10.8%	59.9%
White – British	54	17.1%	77.0%
Other*	48	23.0%	100.0%
Presence of cirrhosis			
No	168	53.3%	53.3%
Yes – Fibroscan diagnosis	134	42.5%	95.9%
Yes – Histological diagnosis	13	4.1%	100.0%
Child–Pugh score			
A	298	94.6%	94.6%
B	12	3.8%	98.4%
C	5	1.6%	100.0%
Primary liver disease aetiology for EDC surveillance			
Cryptogenic – Cirrhotic (Fibroscan)	2	0.6%	0.6%
Cryptogenic – Cirrhotic (Histology)	2	0.6%	1.2%
HBV – Non-cirrhotic	162	51.4%	52.7%
HBV – Cirrhotic (Fibroscan)	8	2.5%	55.2%
HBV – Cirrhotic (Histology)	1	0.3%	55.6%
Alcohol-related liver disease – Cirrhotic (Fibroscan)	35	11.1%	66.7%
Alcohol-related liver disease – Cirrhotic (Histology)	1	0.3%	67.0%
Autoimmune – Cirrhotic (Fibroscan)	1	0.3%	67.3%
Haemochromatosis – Cirrhotic (Fibroscan)	1	0.3%	67.6%
Haemochromatosis – Cirrhotic (Histology)	1	0.3%	67.9%
NAFLD/MASLD – Non-cirrhotic	2	0.6%	68.6%
NAFLD/MASLD – Cirrhotic (Fibroscan)	28	8.9%	77.5%
NAFLD/MASLD – Cirrhotic (Histology)	6	1.9%	79.4%
Post SVR HCV – Non-cirrhotic	4	1.3%	80.6%
Post SVR HCV – Cirrhotic (Fibroscan)	59	18.7%	99.4%
Post SVR HCV – Cirrhotic (Histology)	2	0.6%	100.0%
Smoking status			
Current	42	13.3%	13.3%
Ex-smoker	31	9.8%	23.2%
Never smoked	190	60.3%	83.5%
Not known	52	16.5%	100.0%
Alcohol intake			
14–21 units per week	12	3.8%	3.8%
<14 units per week	53	16.8%	20.6%
>21 units per week	51	16.2%	36.8%
No alcohol use	155	49.2%	86.0%
Unknown	44	14.0%	100.0%
LSOA data index of multiple deprivation rank (IMD)			
1	5	1.6%	1.6%
2	89	28.3%	29.9%
3	81	25.8%	55.7%
4	41	13.1%	68.8%
5	34	10.8%	79.6%
6	27	8.6%	88.2%
7	11	3.5%	91.7%
8	7	2.2%	93.9%
9	12	3.8%	97.8%
10	7	2.2%	100.0%

⁎ Ethnicity – for all other ethnicities, please see Supplementary Appendix.

**Fig. 1:**
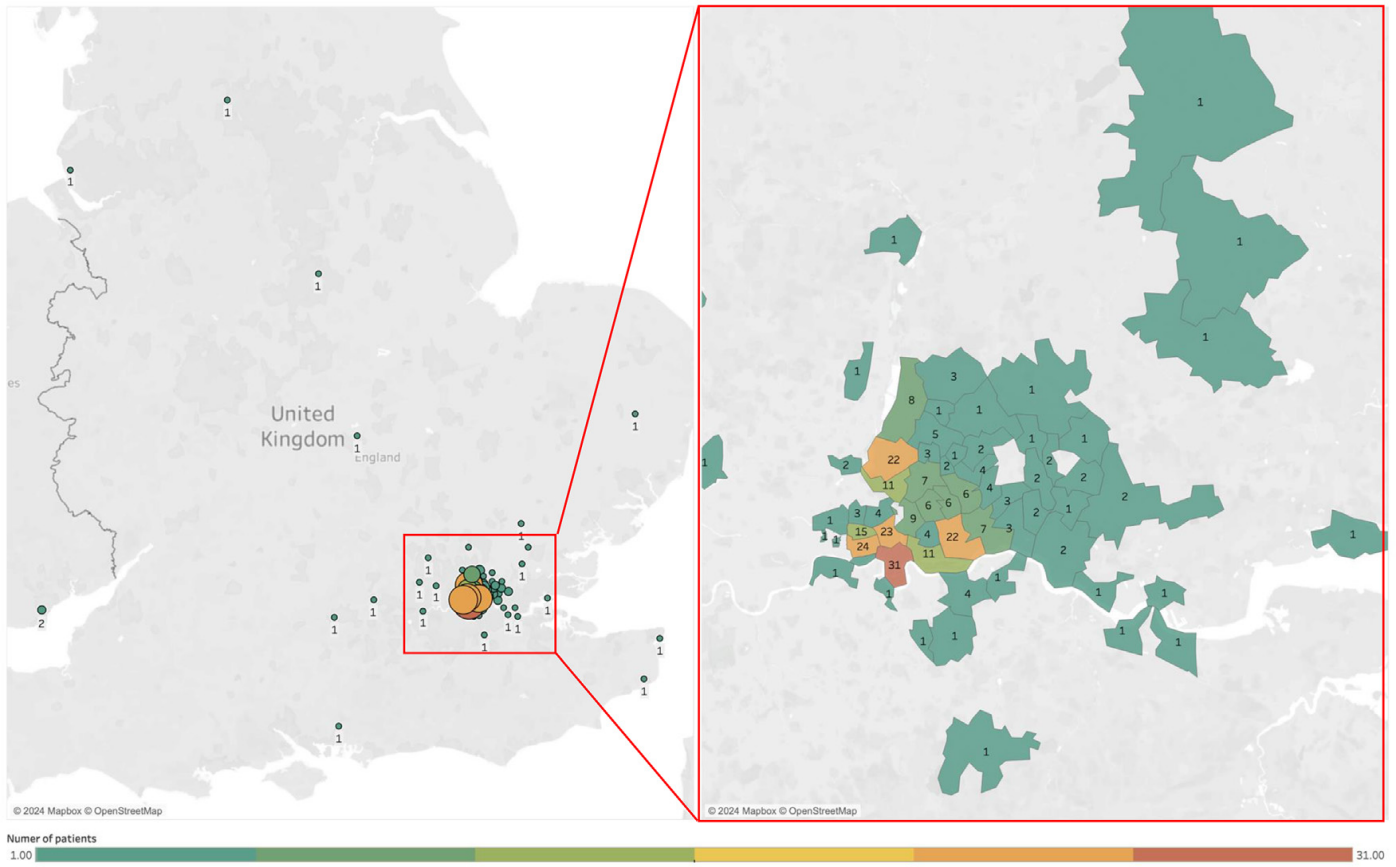
Location and number of patients attending to the early diagnostic centre.

The patients were primarily of Black or Black-British-African background (64–20.3%) followed by White British (54–17.1%) and Asian or Asian British–Bangladeshi (42–13.3%) (Table 1, Supplementary Table 1). When categorised by country of origin, most were of UK White background (67–21.3%), followed by Bangladesh (46–14.6%) and Pakistan (30–9.5%) (Supplementary Table 1). The majority were domiciled in South England, primarily East London (Fig. 1).

From a socio-economic perspective, 68.8% of patients were from a deprived background, with 206 patients of LSOA IMD rank ≤ 4 (Table 1). A majority did not smoke (190, 60.3%) and were teetotal (155, 49.2%). The primary liver aetiology was HBV (171, 54.3%), followed by post-SVR HCV (65, 20.6%), ALD (36, 11.4%), NAFLD/MASLD (36, 11.4%), cryptogenic (4, 1.3%), haemochromatosis (2, 0.6%) with one autoimmune patient (0.3%). Cirrhosis was not present in 186 (53.3%) patients and the majority (298, 94.6%) had compensated liver disease (Child–Pugh A). Of the HCV patients, the majority were genotype 3a (24, 37.5%), with 93% (66/71) receiving treatment and all (71, 100%) achieving SVR (Supplementary Table 1).

Laboratory investigations showed a mean AFP of 3.54, bilirubin 9.95, ALT 25.45, ALP 84.12, AST 28.50, albumin 44.78, platelets 219.19. This corresponded to a mean aMAP score of 50.38 for 313 patients (Supplementary Table 2).

### EDC and general hepatology review

Patients were reviewed for a longer duration in GH (1,486 days per patient) compared to the EDC (291 days per patient) (Table 2) with 2,486 and 465 total appointments booked in GH and EDC, respectively.

**Table 2:** Number of appointments and DNAs at general hepatology (GH) and early diagnostic centre (EDC).

	Mean	Sum	SD	Minimum	Maximum
Pre-EDC general hepatology (GH) clinic					
All appointments					
Number of days reviewed in non-EDC setting	1,486.60	468,911	543.01	210	5,151
Attended (n)	6.00	1,890 (76.03%)	2.49	1	12
DNAs (n)	1.89	596 (23.97%)	2.05	0	12
Virtual appointments*					
Attended (n)	3.66	1,154 (77.29%)	2.32	0	11
DNA (n)	1.07	339 (22.71%)	1.39	0	8
Face-to-face appointments⁎⁎					
Attended (n)	2.12	667 (91.62%)	1.52	0	8
DNAs (n)	0.19	61 (8.38%)	0.55	0	4
Early diagnostic centre (EDC)					
Total days reviewed in EDC	291.33	91,770	98.28	135	1,055
Number of days from EDC referral to review	125.48	39,526	93.38	0	865
Attended (n)	1.24	392 (84.30%)	0.72	0	3
DNAs (n)	0.23	73 (15.70%)	0.54	0	3

Table showing outcomes for the 315 patients.

⁎ 14 patients did not have virtual appointments arranged.

⁎⁎ 21 patients did not have face-to-face appointments arranged.

EDC attendance was improved compared to GH, with a higher total number of DNAs in the GH clinic (596, 23.97%) compared to the EDC (73, 15.70%) (*p* < 0.001) (Table 2). Each patient individually missed 21.67% of their booked GH clinic appointments compared to 13.52% of booked EDC appointments.

Before the initiation of the EDC, 1,493 virtual appointments were booked in GH clinics with 728 face-to-face appointments. Of these, 339 (22.70%) virtual appointments were not attended compared to 61 (8.38%) face-to-face appointments (Table 2). The number of days between reviews in GH clinics ranged from 181 to 259 days (Supplementary Table 3).

For GH clinics attendance, out of 315 patients, 262 (83.3%) did not have an appointment within the recommended HCC surveillance window. Similarly, 119 (37.8%) did not have a scan within the window, and 120 (38.1%) did not have blood tests within the window. Consequently, only 161 (51.1%) of the 315 patients had an ideal HCC assessment within the recommended period for HCC surveillance. Of the remaining 154 patients (48.9%), 101 (32.1%) had a sub-optimal assessment, 34 (10.8%) had an optimal assessment, and 19 (6.0%) had a poor HCC assessment within the recommended period (Table 3).

**Table 3:** HCC surveillance assessment at general hepatology (GH) and early diagnostic centre EDC).

Category	Counts	% of Total	Cumulative %
General hepatology clinic HCC surveillance assessment			
Appointment within window or not			
No	53	16.8%	16.8%
Yes	262	83.2%	100.0%
Scan within window or not			
No	119	37.8%	37.8%
Yes	196	62.2%	100.0%
Blood tests within window or not			
No	120	38.1%	38.1%
Yes	195	61.9%	100.0%
Ideal, optimal, sub-optimal or poor HCC assessment			
Ideal	161	51.1%	51.1%
Poor	19	6.0%	57.1%
Sub-optimal	101	32.1%	89.2%
Optimal	34	10.8%	100.0%
EDC clinic HCC assessment			
Appointment within window or not			
Yes	315	100.0%	100.0%
Scan within window or not			
Yes	315	100.0%	100.0%
Blood tests within window or not			
No	1	0.32%	0.32%
Yes	314	99.68%	100.0%
Ideal, optimal, sub-optimal or poor HCC assessment			
Ideal	314	99.68%	99.78%
Optimal	1	0.32%	100%

Note – as all EDC patients required blood tests and USS being performed on referral to the EDC, all parameters (appointment, scan and bloods) were within the window during the 17-month study period except for one patient who did not have bloods performed within the window.

For the EDC, 314 (99.7%) had an ideal assessment within the recommended HCC surveillance window, with one (0.3%) having optimal HCC assessment over the 17 months of EDC follow-up (Table 3).

### DNA rates secondary to COVID

The highest number of DNAs occurred during the post-COVID period. In GH clinics there were 55 DNAs for 216 patients during the pre-COVID period, 146 DNAs for 257 patients during COVID and 401 DNAs for 315 patients post-COVID; compared to 73 DNAs during the EDC. The consultations during COVID were remote, similar to the EDC clinic. This equates to 0.26 DNAs per patient pre-COVID, 0.57 during COVID and 1.27 per patient post-COVID; compared to 0.23 DNAs per patient during the period of EDC review (Supplementary Table 4).

### Factors associated with incomplete HCC surveillance assessment

In a binomial logistic regression model (Supplementary Table 5), the factors which showed more likelihood of incomplete HCC assessment were female gender (OR 1.47), current smoker (OR 1.06), alcohol intake (OR 1.88), Child–Pugh score B (OR 1.62). However, these were not statistically significant, with the OR confidence intervals crossing the threshold, indicating no significant effect on the completeness of the HCC surveillance assessment.

### Estimates of travel costs and patient journey times

#### Crude estimates of travel cost and time to patient

EDC travel costs were lower; the total travel cost of attending appointments was £3,249 compared to £6,510–£9,765 (lower estimate – upper estimate) for GH clinics. This equates to a crude cost saving of £3,261–£6,516 for attending appointments at the EDC compared to GH clinics (Table 4).

**Table 4:** Estimates of patient travel costs, travel time and DNAs.

	Mean	Total
General hepatology (GH) travel cost and travel time estimates		
Cost for one appointment travel	5.17	£1,627
Total cost (upper estimate)	31.00	£9,765
Total cost (lower estimate)	20.67	£6,510
Time to appointment	41.58	13,097 min
Total time (lower estimate – upper estimate)	83.16–124.73	26,194 - 39,291 min
EDC travel cost and travel time estimates		
Cost for one appointment travel	5.16	£1,625
Total cost	10.31	£3,249
Time to appointment	44.57 min	14,041 min
Total time	89.15 min	28,082 min
Comparison between EDC and GH		
Cost difference in EDC compared to GH (lower estimate – upper estimate)	−10.35 to −20.68	£-3,261 to £-6,516
Time difference in EDC compared to GH (lower estimate – upper estimate)	+5.99 to −35.58	1,888 to 11,209
Total cost of DNAs		
All general hepatology (GH)	£380.30	£119,796
GH virtual appointments	£143.57	£45,226
GH face-to-face appointment	£38.92	£12,261
Early diagnostic centre (EDC)	£37.08	£11,695

The total ToA at the EDC was lower at 28,028 min (around 461 h) compared with 26,194–39,291 min (437–655 h; LE–UE) to the GH clinics. As the number of appointments for the EDC were fewer, this equated to the patients saving a total of 11,209 min (187 h, UE) or spending 1,888 min extra (31 h, LE) on travel to the EDC compared to the GH clinics (Table 4).

#### Crude estimates of cost of DNAs to the healthcare service

Fewer DNAs in the EDC (73) compared to the GH (596), led to a reduction in NHS cost. Utilising the NHS NCC, the estimated EDC cost equates to £11,695 compared to £119,796 in the GH setting (Table 4). There were 342 DNAs in the virtual settings with 64 DNAs in the face-to-face settings, equating to £45,226 loss and £12,261, respectively (Table 4). Of known cancellations, most were patient cancellations (366) with four hospital cancellations.

### Patient outcome

Of 315 patients, one (0.3%) developed HCC during the surveillance period (500 days) with 314 (99.7%) continuing HCC surveillance (Supplementary Table 6) and no patient died during the follow-up period. This patient had a high-risk aMAP score of 60.12, staged A (early stage) under the criteria with referral time from onset to HCC treatment of 10 days and is currently undergoing HCC treatment.

### Socio-economic status and non-attendance

On stratification by socio-economic status, most of the DNAs were by patients of lower socio-economic status (IMD rank 2-3, 214 DNAs). This was similar for all DNAs, even when average and total ToA was factored in, with 160 and 171 DNAs by IMD rank 2–3 with average time and total ToA of less than 40 min and 120 min, respectively (Supplementary Fig. 1). For HCC surveillance assessment by LSOA status, IMD rank 3 had both the most patients with poor and ideal HCC surveillance assessments (10 (52.63%) and 45 (27.95%), respectively) (Supplementary Figs. 2–3).

### Ethnicity and non-attendance

Ethnicity did not significantly impact the rates of DNAs, median missed appointments or ideal HCC assessments. (Supplementary Figs. 4–6). The highest number of DNAs were from Black or Black British-African background (160–27.87%), followed by White British (78–13.59%) and British-Bangladeshis (72–12.54%)**.**

Of ethnicities with adequate numbers of patients, British-Bangladeshis had the fewest patients with ideal HCC assessments (47.06%), followed by Black or Black British-African (50%) compared to British-Pakistanis (64%) and White British (53.7%).

## Discussion

In our single-centre study, we observed higher attendance for HCC surveillance, with significant cost and time savings for patients and healthcare providers following the establishment of the EDC. With nearly all EDC patients reviewed in the recommended time-frame, this centralised clinic model enhanced efficiency and streamlining of the HCC surveillance process, particularly following the COVID pandemic. Similar observations have been seen in screening for breast cancer,^15^ cervical cancer^16^^,^^17^ and cancers of unknown aetiology.^18^

Most patients in our study are from deprived backgrounds and predominantly male. A majority were teetotal, in contrast to other studies,^19^ possibly reflecting the religious background of our population.^20^ The patient cohort primarily included individuals with non-cirrhotic HBV, post-SVR HCV, and NAFLD, with a notable representation of White British, Black African, Bangladeshi and Pakistani ethnicities.

Tthe EDC’s streamlined approach is associated with significantly reduced costs and patient travel time, with higher patient compliance, and thus of particular benefit to patients from deprived areas. These findings were observed within a highly active inner-city capital location which has a unique healthcare burden and complex challenges,^21^ underscoring the robustness and applicability of the intervention in complex urban environments. Akin to other cancer studies,^22^, ^23^, ^24^, ^25^, ^26^ indirect cost and time savings of utilising may help reduce patients’ financial burden and increase uptake of HCC surveillance.

On stratification by COVID time periods, we observed a higher number of DNAs post-COVID compared to both pre-COVID and COVID time periods. One factor may be that patients were vulnerable due to immunosuppression and reluctant to attend hospital settings after the COVID pandemic, similar to patients attending for screening for breast,^27^, ^28^, ^29^ cervical^30^ and colorectal cancer,^30^ for routine outpatient appointments for people living with HIV^31^ and general outpatient services in both adult and paediatric settings.^32^^,^^33^ In our study, with the utilisation of the EDC, the DNA rates were reduced to pre-COVID pandemic levels.

Over 99.7% received an ideal assessment, as recommended by global liver society guidelines. This is contradictory to other studies where, even with telehealth solutions, HCC surveillance was deemed to be sub-optimal to guidelines^8^^,^^34^, ^35^, ^36^, ^37^ with a meta-analysis demonstrating an uptake of HCC surveillance of 24% in the highest risk group.^38^ This may be due to MEH being a separate cold-diagnostic site, which eases access to routine tests in quieter, less crowded settings. With its enhanced efficiency model and by streamlining services, the EDC model may relieve pressure on acute hospitals. It may also support public health by improving access to preventive diagnostics and creating a more resilient healthcare system.

DNAs were most frequent among patients with higher socio-economic deprivation, with ethnic minority patients having the fewest ideal HCC assessments, similar to other studies in both Global North^39^, ^40^, ^41^, ^42^ and Global South^43^, ^44^, ^45^, ^46^ countries.

For the average time and cost to patients, White and Asian patients travelled the furthest, followed by British Pakistanis. The cost was highest for British Africans, Chinese people and British Pakistanis (Supplementary Fig. 7). When stratified by ethnic heritage, British Pakistanis had the highest cost and travel time, followed by people of African heritage. Despite the higher cost and time, 66.67% of British Pakistanis had an ideal HCC assessment, signifying other factors deterring ethnic minorities on attending for HCC assessments. When reviewed by socioeconomic deprivation (Supplementary Fig. 1 and Supplementary Fig. 3), although ToA was lowest for the most deprived patients, these had the highest number of DNAs indicating other unexplored barriers to HCC screening attendance.

## Limitations

The primary limitation of our study lies in its observational nature; while our findings offer valuable insights, they should be interpreted with caution due to the inherent limitations associated with observational research.

As EDC-only patients were captured, there may be patients who dropped out of conventional follow-up, resulting in high selection bias. However, the EDC is a key referral point for the local team to engage patients who have previously missed multiple appointments.

Other limitations include several assumptions regarding patient travel behaviours and cost considerations as detailed in Supplementary Appendix 5. As these are crude cost estimates, further research is warranted, including a cost-modelling study.

Finally, although more patients received an ideal assessment in the EDC, the total days reviewed in the EDC were significantly lower compared to GH clinics and further research is needed to ensure its transferability over a longer time period.

## Conclusion

The EDC model allowed more patients to complete a comprehensive, timely assessment for liver cancer surveillance and demonstrated significant economic efficiency, with a lower cost of DNAs compared to the general hepatology clinics. These findings highlight the integrated clinic model’s potential to improve HCC surveillance efficiency, reduce healthcare costs and enhance patient compliance, particularly for socio-economically disadvantaged and ethnic minority populations.

## Ethical approval and consent to participate

The project was an audit of a newly implemented NHS service and did not deviate from standard of care. All data were anonymised and patient information handled in compliance with GDPR and NHS information governance protocols. No specific ethics approval was needed under the guidance of the Health Research Authority guidelines.

## Data availability statement

The data that support the findings in this study are available from the corresponding author upon reasonable request.

## CRediT authorship contribution statement

**Hamzah Z. Farooq:** Writing – review & editing, Writing – original draft, Visualization, Validation, Software, Resources, Project administration, Methodology, Investigation, Funding acquisition, Formal analysis, Data curation, Conceptualization. **Kevin Galono:** Writing – review & editing, Data curation. **Leila Reid:** Writing – review & editing. **Janet Dearden:** Writing – review & editing, Supervision. **Graham R. Foster:** Writing – review & editing, Writing – original draft, Validation, Supervision, Resources, Project administration, Methodology, Investigation, Funding acquisition, Conceptualization.

## Declaration of competing interest

The authors declare that they have no known competing financial interests or personal relationships that could have appeared to influence the work reported in this paper.

## Appendix Supplementary materials

Image, application 1

Image, application 2
