# Supplementary material for: Evaluating hepatocellular carcinoma (HCC) surveillance through an early diagnostic centre: An implementation science approach at a tertiary hepatology centre in England
Source: Clin Med (Lond). 2025 Nov 13;26(1):100531. doi: 10.1016/j.clinme.2025.100531 (PMC12753212; doi:10.1016/j.clinme.2025.100531)
Supplement: Supplementary file 1 [file mmc1.docx]

**Supplementary Appendix**

**Supplementary table 1 – Additional demographics**

| **Category** | **Counts** | **Percent of total** | **Cumulative Percentage** |
| --- | --- | --- | --- |
| **Other Ethnicity** |  |  |  |
| Asian or Asian British - Indian | 9 | 2.9 % | 2.9% |
| Black or Black British - Caribbean | 2 | 0.6 % | 3.5% |
| Black - Any Other Black Background | 13 | 4.1 % | 7.6% |
| Mixed - White and Asian | 1 | 0.3 % | 7.9% |
| Mixed - White and Black African | 1 | 0.3 % | 8.2% |
| Other - Any Other Ethnic Group | 18 | 5.7 % | 13.9% |
| Other - Chinese | 14 | 4.4 % | 18.3% |
| Other - Not Stated | 3 | 1.0 % | 19.3% |
| Patient Refused | 8 | 2.5 % | 21.8 |
| White - Irish | 3 | 1.0 % | 22.8% |
| **Ethnicity Heritage** |  |  |  |
| Afghanistan | 1 | 0.3 % | 18.7 % |
| Albania | 1 | 0.3 % | 19.0 % |
| Angola | 1 | 0.3 % | 19.4 % |
| Bangladesh | 18 | 5.7 % | 25.1 % |
| Barbados | 2 | 0.6 % | 25.7 % |
| Bosnia | 1 | 0.3 % | 26.0 % |
| Brazil | 1 | 0.3 % | 26.3 % |
| Burma | 2 | 0.6 % | 27.0 % |
| Cameroon | 18 | 5.7 % | 32.7 % |
| Cape Verde | 1 | 0.3 % | 33.0 % |
| China | 12 | 3.8 % | 36.8 % |
| Congo | 1 | 0.3 % | 37.1 % |
| Democratic Republic of Congo | 3 | 1.0 % | 38.1 % |
| Ethiopia | 1 | 0.3 % | 38.4 % |
| Gambia | 1 | 0.3 % | 38.7 % |
| Ghana | 1 | 0.3 % | 39.0 % |
| Guyana | 5 | 1.6 % | 40.6 % |
| India | 1 | 0.3 % | 41.0 % |
| Indonesia | 14 | 4.4 % | 45.4 % |
| Ireland | 3 | 1.0 % | 46.3 % |
| Italy | 30 | 9.5 % | 55.9 % |
| Italy/Bangladesh | 2 | 0.6 % | 56.5 % |
| Japan | 6 | 1.9 % | 58.4 % |
| Lithuania | 1 | 0.3 % | 58.7 % |
| Netherlands | 1 | 0.3 % | 41.0 % |
| Nigeria | 14 | 4.4 % | 45.4 % |
| Not known | 3 | 1.0 % | 46.3 % |
| Pakistan | 30 | 9.5 % | 55.9 % |
| Philippines | 2 | 0.6 % | 56.5 % |
| Poland | 6 | 1.9 % | 58.4 % |
| Portugal | 1 | 0.3 % | 58.7 % |
| Romania | 4 | 1.3 % | 60.0 % |
| Russian | 2 | 0.6 % | 60.6 % |
| Senegal | 3 | 1.0 % | 61.6 % |
| Sierra Leone | 3 | 1.0 % | 62.5 % |
| Somalia | 16 | 5.1 % | 67.6 % |
| Sri Lanka | 2 | 0.6 % | 68.3 % |
| Taiwan | 1 | 0.3 % | 68.6 % |
| Tunisia | 1 | 0.3 % | 68.9 % |
| Turkey | 3 | 1.0 % | 69.8 % |
| UK - Afro-Caribbean | 1 | 0.3 % | 70.2 % |
| UK - White | 67 | 21.3 % | 91.4 % |
| Uganda | 2 | 0.6 % | 92.1 % |
| Unknown | 2 | 0.6 % | 92.7 % |
| Unknown - Africa | 16 | 5.1 % | 97.8 % |
| Vietnam | 5 | 1.6 % | 99.4 % |
| White | 1 | 0.3 % | 99.7 % |
| Zimbabwe | 1 | 0.3 % | 100.0 % |
|  |  |  |  |
| **HCV Genotype** |  |  |  |
| 1 | 2 | 3.1 % | 3.1 % |
| 1a | 9 | 14.1 % | 17.2 % |
| 1b | 6 | 9.4 % | 26.6 % |
| 3 | 9 | 14.1 % | 40.6 % |
| 4 | 2 | 3.1 % | 43.8 % |
| Not known | 7 | 10.9 % | 54.7 % |
| 3a | 24 | 37.5 % | 92.2 % |
| 3b | 3 | 4.7 % | 96.9 % |
| 4b | 1 | 1.6 % | 98.4 % |
| 3g | 1 | 1.6 % | 100.0 % |
| **Received HCV treatment** |  |  |  |
| No | 5 | 7.0 % | 7.0 % |
| Yes | 66 | 93.0 % | 100.0 % |
| **Achieved SVR** |  |  |  |
| Yes | 70 | 100.0 % | 100.0 % |
| **Concurrent liver disease** |  |  |  |
| Alcohol-related liver disease | 5 | 1.6 % | 1.6 % |
| Haemochromotosis, MASLD | 1 | 0.3 % | 1.9 % |
| Methotrexate-induced cirrhosis | 1 | 0.3 % | 2.2 % |
| NAFLD/MASLD | 16 | 5.1 % | 7.3 % |
| Nil | 290 | 92.1 % | 99.4 % |
| Post SVR HCV | 2 | 0.6 % | 100.0 % |

**Supplementary table 2 – Blood parameters, BMI and aMAP scores of cohort**

|  | N | Missing | Mean | Median | Mode | SD | IQR | Minimum | Maximum |
| --- | --- | --- | --- | --- | --- | --- | --- | --- | --- |
| Age at appointment | 315 | 0 | 53.93 | 52 | 51.00 | 11.75 | 16.50 | 21 | 90 |
| BMI | 247 | 68 | 28.85 | 27.56 | 28.00 | 7.22 | 6.99 | 15.32 | 83.3 |
| AFP | 311 | 4 | 3.54 | 2.20 | 2.00 | 6.56 | 1.40 | 1.80 | 110.0 |
| Total Bilirubin | 314 | 1 | 9.95 | 8.00 | 6.00 | 6.64 | 5.00 | 3.00 | 47.0 |
| ALT | 314 | 1 | 25.45 | 22.00 | 14.00 | 14.44 | 15.00 | 5.00 | 135.0 |
| ALP | 314 | 1 | 84.12 | 76.00 | 82.00 | 34.85 | 31.00 | 13.00 | 374.0 |
| AST | 311 | 4 | 28.50 | 24.00 | 21.00 | 19.23 | 10.00 | 11.00 | 223.0 |
| Albumin | 311 | 4 | 44.78 | 45.00 | 46.00 | 3.72 | 4.00 | 28.00 | 78.0 |
| Platelets | 313 | 2 | 219.19 | 215.00 | 218.00 | 70.64 | 90.00 | 36.00 | 434.0 |
| aMAP score | 313 | 2 | 50.38 | 50.29 | 27.91 | 8.45 | 11.96 | 27.91 | 75.0 |
| ᵃ More than one mode exists, only the first is reported  Some patients did not have full blood investigations performed and hence some values are not 315 | | | | | | | | | |

**Supplementary table 3 – Time interval between reviews at General Hepatology clinics**

|  | N | Missing | Mean | Median | Mode | Sum | SD | Variance | IQR | Range | Minimum | Maximum |
| --- | --- | --- | --- | --- | --- | --- | --- | --- | --- | --- | --- | --- |
| Days to Full assessment in GH Clinics | 315 | 0 | 208 | 163 | 51.0* | 65618 | 225.2 | 50695 | 169.5 | 1540 | 5 | 1545 |
| Days between GH Review: | | | | | | | | | | | | |
| 1^st^ to 2^nd^ review | 303 | 12 | 227 | 182 | 182.0 | 68796 | 157.1 | 24691 | 126.0 | 1272.0 | 10.00 | 1282 |
| 2^nd^ to 3^rd^ review | 279 | 36 | 259 | 195 | 182.0 | 72302 | 170.4 | 29043 | 172.0 | 1400.0 | 14.00 | 1414 |
| 3^rd^ to 4^th^ review | 254 | 61 | 241 | 196 | 182.0 | 61236 | 128.7 | 16573 | 120.3 | 727 | 0.00 | 721 |
| 4^th^ to 5^th^ review | 222 | 93 | 229 | 189 | 182.0 | 50922 | 109.9 | 12074 | 57.5 | 736.0 | 13.00 | 749 |
| 5^th^ to 6^th^ review | 182 | 133 | 211 | 189 | 182.0 | 38361 | 90.8 | 8236 | 30.8 | 678.0 | 15.00 | 693 |
| 7^th^ to 8^th^ review | 142 | 173 | 207 | 184 | 182.0 | 29428 | 93.8 | 8799 | 38.3 | 658.0 | 28.00 | 686 |
| 8^th^ to 9^th^ review | 94 | 221 | 195 | 182 | 182.0 | 18347 | 82.9 | 6876 | 50.5 | 539.0 | 6.00 | 545 |
| 9^th^ to 10^th^ review | 41 | 274 | 181 | 182 | 182.0 | 7421 | 60.4 | 3653 | 42.0 | 325.0 | 10.00 | 335 |
| 10^th^ to 11^th^ review | 15 | 300 | 194 | 182 | 175.0 | 2916 | 59.3 | 3516 | 28.0 | 259.0 | 105.00 | 364 |
| 11^th^ to 12^th^ review | 6 | 309 | 185 | 183 | 91.0* | 1108 | 61.0 | 3722 | 30.3 | 189.0 | 91.00 | 280 |
| 12^th^ to 13^th^ review | 2 | 313 | 186 | 186 | 175.0* | 371 | 14.8 | 221 | 10.5 | 21.0 | 175.00 | 196 |
| 13^th^ to 14^th^ review | 0 | 315 | Nil | Nil | Nil | Nil | Nil | Nil | Nil | Nil | Nil | Nil |
| **More than one mode exists, only the first is reported* | | | | | | | | | | | | |

**Supplementary table 4 – DNAs of patients in general hepatology during the pre-COVID, COVID and post-COVID time periods, compared to EDC**

|  | Number of days during defined period | Number of patients | Missing | Mean | Median | Mode | Sum | SD | Minimum | Maximum |
| --- | --- | --- | --- | --- | --- | --- | --- | --- | --- | --- |
| DNAs in General Hepatology | | | | | | | | | | |
| Pre-COVID | 447 | 216 | 99* | 0.26 | 0 | 0.00 | 55 | 0.61 | 0 | 3 |
| COVID | 483 | 257 | 58** | 0.57 | 0 | 0.00 | 146 | 0.90 | 0 | 4 |
| Post- COVID | 470 | 315 | 0 | 1.27 | 1 | 0.00 | 401 | 1.51 | 0 | 8 |
| DNAs in EDC | | | | | | | | | | |
| EDC | 501 | 315 | 0 | 0.23 | 0 | 0.00 | 73 | 0.54 | 0 | 3 |
|  | | | | | | | | | | |
| Note  Definition of eras: Pre-COVID is from 1^st^ January 2019 until 23^rd^ March 2020 (First day of UK-wide lockdown); COVID era is from 23^rd^ March 2020 until 19^th^ July 2021 (the end of all lockdown restrictions in the UK); Post COVID is from 20^th^ July 2021 until the start of the EDC on the 1^st^ 0f November 2022).  * 99 patients had their first review for HCC surveillance after the pre-COVID era  ** 58 patients had their first review for HCC surveillance after the COVID era | | | | | | | | | | |

**Supplementary table 5 – Binomial logistic regression model on complete HCC surveillance assessment (Ideal HCC surveillance) or incomplete HCC surveillance assessment (Optimal, Sub-optimal, Poor HCC surveillance)**

| Model Co-efficients – Complete assessment (Ideal) or Incomplete assessment (Optimal, Sub-optimal, Poor) | | | | | | 95% Confidence Interval | |
| --- | --- | --- | --- | --- | --- | --- | --- |
| Predictor | **Estimate** | **SE** | **Z** | **p** | **Odds ratio** | **Lower** | **Upper** |
| Intercept | -2.47289 | 2.1058 | -1.17433 | 0.240 | 0.0843 | 0.00136 | 5.23 |
| Gender – Reference (R): Male |  |  |  |  |  |  |  |
| Female | 0.38730 | 0.3455 | 1.12102 | 0.262 | **1.4730** | 0.74838 | 2.90 |
| Smoking status- (R: Never smoked) |  |  |  |  |  |  |  |
| Current smoker | 0.05854 | 0.4834 | 0.12110 | 0.904 | **1.0603** | 0.41112 | 2.73 |
| Ex-Smoker | -0.62455 | 0.5085 | -1.22821 | 0.219 | 0.5355 | 0.19766 | 1.45 |
| Not known | 0.29280 | 0.5616 | 0.52139 | 0.602 | 1.3402 | 0.44580 | 4.03 |
| Alcohol intake – (R: No alcohol use) |  |  |  |  |  |  |  |
| 14-21 Units per week | 0.63012 | 0.8365 | 0.75327 | 0.451 | **1.8778** | 0.36444 | 9.68 |
| <14 Units per week | 0.48388 | 0.4093 | 1.18220 | 0.237 | **1.6224** | 0.72735 | 3.62 |
| >21 Units per week | 0.32119 | 0.7602 | 0.42253 | 0.673 | **1.3788** | 0.31077 | 6.12 |
| Unknown | -0.25756 | 0.6001 | -0.42921 | 0.668 | 0.7729 | 0.23842 | 2.51 |
| Index of Multiple Deprivation Rank – (R: IMD Rank 10) |  |  |  |  |  |  |  |
| 1 | 0.51466 | 1.5041 | 0.34216 | 0.732 | **1.6731** | 0.08774 | 31.90 |
| 2 | 0.40305 | 0.9011 | 0.44729 | 0.655 | **1.4964** | 0.25588 | 8.75 |
| 3 | 0.01616 | 0.9040 | 0.01788 | 0.986 | **1.0163** | 0.17280 | 5.98 |
| 4 | -0.09894 | 0.9558 | -0.10352 | 0.918 | 0.9058 | 0.13913 | 5.90 |
| 5 | 0.09972 | 0.9463 | 0.10537 | 0.916 | **1.1049** | 0.17290 | 7.06 |
| 6 | 0.59357 | 0.9399 | 0.63156 | 0.528 | **1.8104** | 0.28693 | 11.42 |
| 7 | -0.92705 | 1.1678 | -0.79384 | 0.427 | 0.3957 | 0.04012 | 3.90 |
| 8 | 0.77489 | 1.2101 | 0.64033 | 0.522 | **2.1704** | 0.20251 | 23.26 |
| 9 | -0.16282 | 1.1605 | -0.14030 | 0.888 | 0.8497 | 0.08738 | 8.26 |
| Ethnicity – (R: White-British) |  |  |  |  |  |  |  |
| Asian - Any Other Asian Background | 0.09278 | 0.6531 | 0.14207 | 0.887 | **1.0972** | 0.30507 | 3.95 |
| Asian or Asian British - Bangladesh | 0.74376 | 0.6030 | 1.23353 | 0.217 | **2.1038** | 0.64532 | 6.86 |
| Asian or Asian British - Indian | 0.23936 | 0.9444 | 0.25344 | 0.800 | **1.2704** | 0.19956 | 8.09 |
| Asian or Asian British - Pakistani | -0.07872 | 0.6505 | -0.12101 | 0.904 | 0.9243 | 0.25828 | 3.31 |
| Black - Any Other Black Background | 0.32453 | 0.8160 | 0.39773 | 0.691 | **1.3834** | 0.27951 | 6.85 |
| Black or Black British - African | 0.64645 | 0.6105 | 1.05896 | 0.290 | **1.9087** | 0.57694 | 6.31 |
| Black or Black British - Caribbean | 17.02299 | 1696.4058 | 0.01003 | 0.992 | 2.47e+7 | 0.00000 | Inf |
| Mixed - White and Asian | 17.76911 | 2399.5448 | 0.00741 | 0.994 | 5.21e+7 | 0.00000 | Inf |
| Mixed - White and Black African | -15.98666 | 2399.5448 | -0.00666 | 0.995 | 1.14e-7 | 0.00000 | Inf |
| Other - Any Other Ethnic Group | 1.19454 | 0.6990 | 1.70882 | 0.087 | **3.3020** | 0.83899 | 13.00 |
| Other - Chinese | 0.13749 | 0.7836 | 0.17545 | 0.861 | 1.1474 | 0.24699 | 5.33 |
| Other - Not Stated | 1.66747 | 1.4170 | 1.17678 | 0.239 | **5.2988** | 0.32964 | 85.17 |
| Patient Refused | 1.73455 | 1.0898 | 1.59162 | 0.111 | **5.6664** | 0.66936 | 47.97 |
| White - Any Other White Background | 0.62197 | 0.5592 | 1.11224 | 0.266 | 1.8626 | 0.62247 | 5.57 |
| White - Irish | 0.75009 | 1.5241 | 0.49215 | 0.623 | **2.1172** | 0.10677 | 41.98 |
| Primary liver disease aetiology for EDC surveillance – (R: Post SVR HCV) |  |  |  |  |  |  |  |
| Cryptogenic | -0.54548 | 1.2945 | -0.42138 | 0.673 | 0.5796 | 0.04584 | 7.33 |
| HBV | -0.50606 | 0.6573 | -0.76988 | 0.441 | 0.6029 | 0.16623 | 2.19 |
| Alcohol-related liver disease | -0.55262 | 0.8252 | -0.66971 | 0.503 | 0.5754 | 0.11419 | 2.90 |
| Autoimmune | -16.99760 | 2399.5449 | -0.00708 | 0.994 | 4.15e-8 | 0.00000 | Inf |
| Haemochromatosis | -1.42684 | 1.7227 | -0.82825 | 0.408 | 0.2401 | 0.00820 | 7.03 |
| NAFLD/MASLD | -0.39028 | 0.5746 | -0.67919 | 0.497 | 0.6769 | 0.21947 | 2.09 |
| Presence of cirrhosis – (R: No cirrhosis) |  |  |  |  |  |  |  |
| Yes - Fibroscan diagnosis | -0.20276 | 0.6167 | -0.32880 | 0.742 | 0.8165 | 0.24381 | 2.73 |
| Yes - Histological diagnosis | 0.37790 | 0.9015 | 0.41919 | 0.675 | **1.4592** | 0.24933 | 8.54 |
| Concurrent liver disease -(R: Alcohol-related liver disease) |  |  |  |  |  |  |  |
| Haemochromatosis and MASLD | 17.65751 | 2399.5456 | 0.00736 | 0.994 | **4.66e+7** | 0.00000 | Inf |
| Methotrexate-induced cirrhosis | -15.58677 | 2399.5451 | -0.00650 | 0.995 | 1.70e-7 | 0.00000 | Inf |
| NAFLD/MASLD | 0.18590 | 1.3183 | 0.14102 | 0.888 | 1.2043 | 0.09091 | 15.95 |
| Nil | 0.96072 | 1.2089 | 0.79474 | 0.427 | **2.6136** | 0.24449 | 27.94 |
| Post SVR HCV | -14.95636 | 1696.0435 | -0.00882 | 0.993 | 3.20e-7 | 0.00000 | Inf |
| AFP | -0.01866 | 0.0227 | -0.82356 | 0.410 | 0.9815 | 0.93887 | 1.03 |
| Childs Pugh Score – (R: Childs-Pugh A) |  |  |  |  |  |  |  |
| B | 0.48799 | 0.7527 | 0.64829 | 0.517 | **1.6290** | 0.37256 | 7.12 |
| C | 16.86667 | 1051.4841 | 0.01604 | 0.987 | **2.11e+7** | 0.00000 | Inf |
| aMAP score | 0.00449 | 0.0280 | 0.16002 | 0.873 | **1.0045** | 0.95080 | 1.06 |
| Age at appointment | 0.01568 | 0.0182 | 0.86034 | 0.390 | **1.0158** | 0.98016 | 1.05 |
| *Note. Estimates represent the log odds of "Complete_Assessment_or_Incomplete_Assessment = Incomplete" vs. "Complete_Assessment_or_Incomplete_Assessment = Complete"* | | | | | | | |

| Model Fit Measures | | | | | | | | | | | | | | | | | | | | | |
| --- | --- | --- | --- | --- | --- | --- | --- | --- | --- | --- | --- | --- | --- | --- | --- | --- | --- | --- | --- | --- | --- |
|  | | | | | | | | | | | | | | | | **Overall Model Test** | | | | | |
| Model | | **Deviance** | | **AIC** | | **BIC** | | **R²_McF_** | | **R²_CS_** | | **R²_N_** | | **R²_T_** | | **χ²** | | **df** | | **p** | |
| 1 |  | 380 |  | 482 |  | 672 |  | 0.112 |  | 0.144 |  | 0.192 |  | 0.133 |  | 48.1 |  | 50 |  | 0.548 |  |
|  | | | | | | | | | | | | | | | | | | | | | |

**Supplementary table 6 – Outcome of cohort**

| Category | Counts | % of Total | Cumulative % |
| --- | --- | --- | --- |
|  |  |  |  |
| Development of HCC | | | |
| No | 314 | 99.7 % | 99.7 % |
| Yes | 1 | 0.3 % | 100.0 % |
|  |  |  |  |
| If developed HCC, staging | | | |
| Stage A (early stage) | 1 | 100 % | 100% |
|  |  |  |  |
| Referral time for HCC treatment from HCC onset | | | |
| 10 days | 1 | 100% | 100% |
|  |  |  |  |
| Outcome | | | |
| Alive with disease (AWD) | 314 | 99.7 % | 99.7 % |
| Cured of liver disease | 1 | 0.3 % | 100.0 % |
|  |  |  |  |
|  |  |  |  |

**Supplementary Appendix 1** - **EDC Description and Referral Criteria**

1. Departmental description and function

The Early Diagnostic Centre at Mile End Hospital is the first of its kind in the country with the aim of improving early diagnosis for patients with conditions that increase their risk of developing cancer. The centre is equipped with endoscopy, phlebotomy, ultrasound, CT scan and MRI.

The centre will be offering tests to patients living with specific conditions who need regular surveillance diagnostics. We will offer a non-acute service, seeing patients with uncomplicated histories who require surveillance exams. Imaging department will particularly cater to patients at risk of developing hepatocellular carcinoma requiring surveillance ultrasound and blood tests.

Referrals will come from one of the three partnership trusts based in the North East Thames Clinical Commissioning Groups. They include Barking, Havering & Redbridge University Hospitals NHS Trust, Bart’s Health NHS Trust and Homerton University Hospital NHS Trust.

Bart’s will be the host trust to deliver the service led by one of the Hepatology consultants. The team will comprise of a radiology consultant, clinical nurse specialist, radiographers, phlebotomist and administrative staff, to provide safe and excellent service.

1. Areas within the department and associated equipment
   1. Reception area

The main reception area is located in the imaging department of Mile End Hospital and has seating for twenty, along with 4 toilets including one for disabled access.

Patients enter and are greeted by the imaging reception staff, who, in turn admit them on the Sectra RIS patient records computer system. They will wait to be called through by one of the radiographers for their ultrasound and will proceed to see the phlebotomists to have their blood examination taken.

- 1. Ultrasound rooms

There are 2 ultrasound rooms in this unit with each ultrasound machine (LOGIQ E10). Patients must have an appointment prior to attending their examination. There are allocated radiographers during operational hours to perform the ultrasound scan.

- 1. Phlebotomy room

This room is located in the consulting room 4 within the imaging department. It is equipped with logistical requirements necessary for blood extraction and one phlebotomist during working hours. This is a walk-in service, and patients are advised to have their bloods taken as per request by the referring clinician.

1. Hours of Operation

Monday to Friday 09.00 – 12.00, 13.00 – 16.00

The ultrasound scan and phlebotomy are open 5 days a week. Our schedulers will schedule their due HCC surveillance and book their ultrasound scan through Sectra RIS. Referring clinicians should generate blood request or specify any additional blood examination the patient requires during the initial referral.

1. Staffing

- 1 Hepatology Consultant
- 1 EDC CNS
- 2 Radiographers
- 1 EDC Admin
- 1 Phlebotomist

1. Inclusion and exclusion criteria for HCC surveillance in EDC

Inclusion criteria:

- Cirrhosis – all causes
- Compensated Childs Pugh A
- NAFLD with > moderate liver fibrosis
- Hepatitis C SVR with advanced fibrosis F5/6 or LSM > 9kPa
- Non-cirrhotic hepatitis B in:
- Family History of HCC
- Asian Men >40 years old
- Asian Women >50 years old
- Black or Black British >20 years old
- White – British or any other white background with PAGE-B score ≥10*

PAGE-B (Platelet, Age, Gender, Hepatitis B) score is based on decade of age (16–29 = 0; 30–39 = 2; 40–49 = 4; 50–59 =6; 60–69 = 8; ≥70 = 10), gender (M = 6; F = 0) and platelet count (≥200,000/μl = 0; 100,000–199,999/μl = 6; <100,000/μl= 9)

Exclusion Criteria:

- Hospital Transport patient
- Multiple co-morbidities
- Childs Pugh B / C cirrhosis
- CHB patients on anti-viral treatment

**Supplementary Appendix 2 – Calculations of patient cost and time to appointment (ToA)**

The cost for one journey was then multiplied by six (three return journeys to the appointment – upper estimate [UE]) or multiplied by four (two return journeys to the appointment – lower estimate [LE]) for RLH and multiplied by two (one combined appointment for bloods, US and return as telephone appointment). Using this cost and time data, we calculated the crude estimated total cost and time of travel to both the GH appointments and EDC appointments and calculated the cost and time difference for patients in the EDC compared to the GH clinic.

**Supplementary Appendix 3 - National Schedule of NHS Costs - Year 2021/22 - NHS trusts and NHS foundation trusts**

| **Outpatient Care** | |  |  |  |  |  |  |  |
| --- | --- | --- | --- | --- | --- | --- | --- | --- |
|  |  |  |  |  |  |  |  |  |
|  |  |  |  |  |  |  |  |  |
| **Department Code** | **Department Description** | **Service Code** | **Service Description** | **Currency Code** | **Currency Description** | **Number of Attendances** | **National Average Unit Cost** | **Total Costs** |
| CL | Consultant Led | 306 | Hepatology Service | WF01A | Non-Admitted Face-to-Face Attendance, Follow-up | 148264 | £200.68 | £29,753,796.19 |
| CL | Consultant Led | 306 | Hepatology Service | WF01B | Non-Admitted Face-to-Face Attendance, First | 47230 | £238.98 | £11,287,082.49 |
| CL | Consultant Led | 306 | Hepatology Service | WF01C | Non-Admitted Non-Face-to-Face Attendance, Follow-up | 97069 | £133.41 | £12,950,091.24 |
| CL | Consultant Led | 306 | Hepatology Service | WF01D | Non-Admitted Non-Face-to-Face Attendance, First | 17010 | £145.93 | £2,482,320.51 |
| CL | Consultant Led | 306 | Hepatology Service | WF02A | Multiprofessional Non-Admitted Face-to-Face Attendance, Follow-up | 12522 | £321.44 | £4,025,015.01 |
| CL | Consultant Led | 306 | Hepatology Service | WF02B | Multiprofessional Non-Admitted Face-to-Face Attendance, First | 3753 | £308.59 | £1,158,132.31 |
| CL | Consultant Led | 306 | Hepatology Service | WF02C | Multiprofessional Non-Admitted Non-Face-to-Face Attendance, Follow-up | 7283 | £287.67 | £2,095,100.96 |
| CL | Consultant Led | 306 | Hepatology Service | WF02D | Multiprofessional Non-Admitted Non-Face-to-Face Attendance, First | 955 | £343.69 | £328,224.95 |
| NCL | Non Consultant Led | 306 | Hepatology Service | WF01A | Non-Admitted Face-to-Face Attendance, Follow-up | 18568 | £198.58 | £3,687,291.29 |
| NCL | Non Consultant Led | 306 | Hepatology Service | WF01B | Non-Admitted Face-to-Face Attendance, First | 5995 | £214.59 | £1,286,492.19 |
| NCL | Non Consultant Led | 306 | Hepatology Service | WF01C | Non-Admitted Non-Face-to-Face Attendance, Follow-up | 7909 | £160.18 | £1,266,834.63 |
| NCL | Non Consultant Led | 306 | Hepatology Service | WF01D | Non-Admitted Non-Face-to-Face Attendance, First | 1021 | £108.10 | £110,366.14 |
| NCL | Non Consultant Led | 306 | Hepatology Service | WF02A | Multiprofessional Non-Admitted Face-to-Face Attendance, Follow-up | 108 | £312.52 | £33,751.91 |
| NCL | Non Consultant Led | 306 | Hepatology Service | WF02B | Multiprofessional Non-Admitted Face-to-Face Attendance, First | 31 | £340.51 | £10,555.83 |
| NCL | Non Consultant Led | 306 | Hepatology Service | WF02C | Multiprofessional Non-Admitted Non-Face-to-Face Attendance, Follow-up | * | * | £1,238.30 |
| NCL | Non Consultant Led | 306 | Hepatology Service | WF02D | Multiprofessional Non-Admitted Non-Face-to-Face Attendance, First | * | * | £53.90 |

**Supplementary Appendix 4 – Definition of COVID time-periods**

The pre-COVID period is defined as from 1st January 2019 to 23rd March 2020, the day the UK entered its first nationwide lockdown. The COVID period spans 23rd March 2020 until 19th July 2021 - end of all lockdown restrictions. The post-COVID period began on 20th July 2021 and extended until the start of the EDC on 1st November 2022.

**Supplementary Appendix 5 – Assumptions for patient travel behaviours and cost-considerations**

It was presumed that patients would opt to walk to the hospital if it was quicker than taking public transport. We also assume that patients would choose public transport over driving due to traffic congestion, limited parking at the hospital site (small number of paid parking bays, majority of spaces are for resident permit holders only with a maximum permitted stay of four hours, at a charge of £3.80 per hour) and time considerations, rather than public transportation costs.

The analysis also assumed peak travel costs rather than off-peak rates, standard train tickets instead of first-class, and bus travel costs rather than higher tube fares within Zone 1 on the London Underground network would be preferred by patients. Furthermore, it was assumed that patients did not incur additional travel expenses if their travel routes coincided with their work commutes within the same region.
