# Supplementary material for: Evaluating hepatocellular carcinoma (HCC) surveillance through an early diagnostic centre: An implementation science approach at a tertiary hepatology centre in England
Source: Clin Med (Lond). 2025 Nov 13;26(1):100531. doi: 10.1016/j.clinme.2025.100531 (PMC12753212; doi:10.1016/j.clinme.2025.100531)
Supplement: Supplementary file 2 [file mmc2.docx]

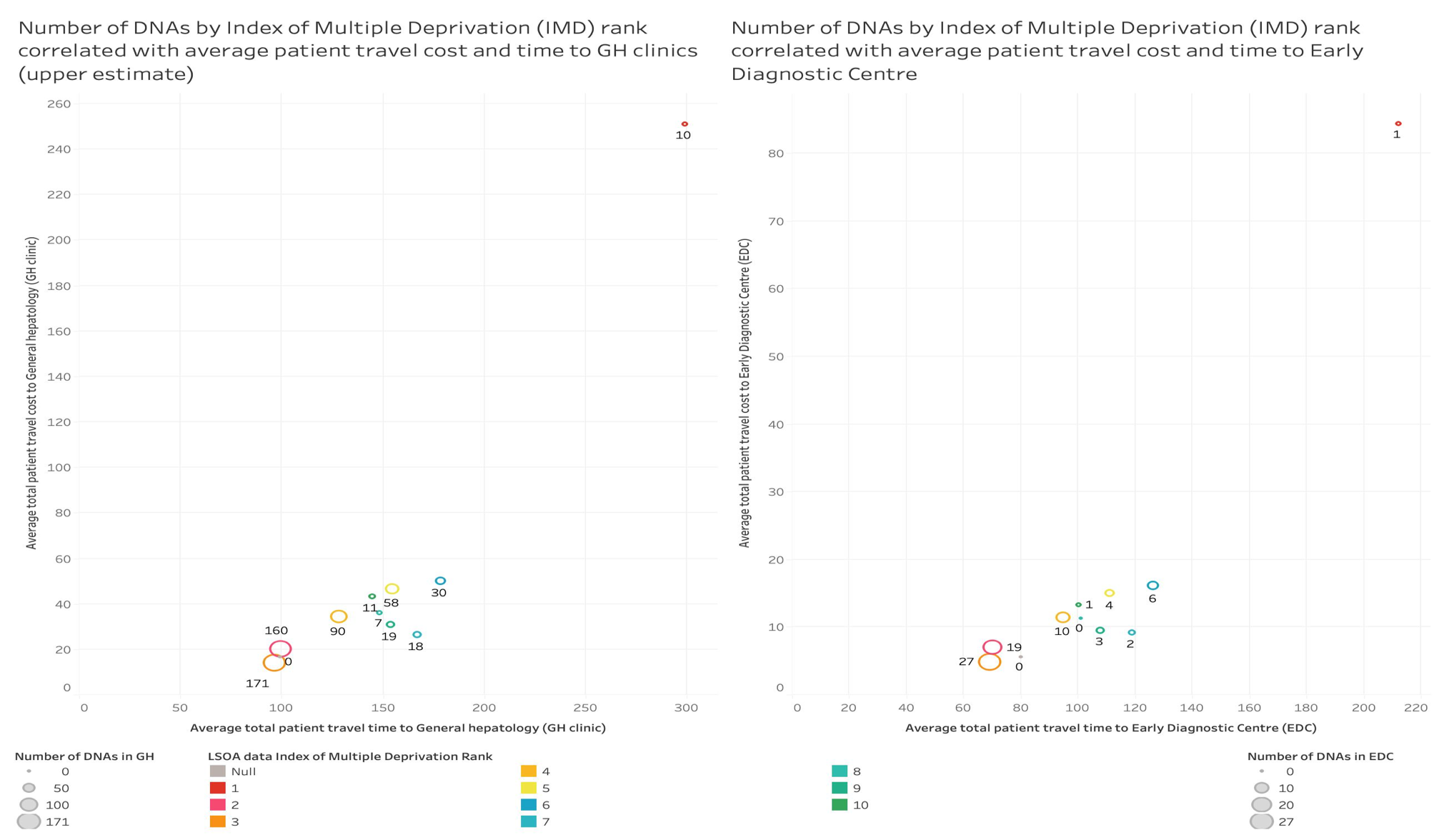


Supplementary Figure 1 – A - Number of DNA stratified by index of multiple deprivation rank (IMD) at the general hepatology clinic, correlated with average total patient cost and time (upper estimate) to the general hepatology clinic (GH); B - Number of DNAs stratified by index of multiple deprivation rank (IMD) at the Early Diagnostic Centre (EDC) clinic, correlated with total average patient time and time to the EDC


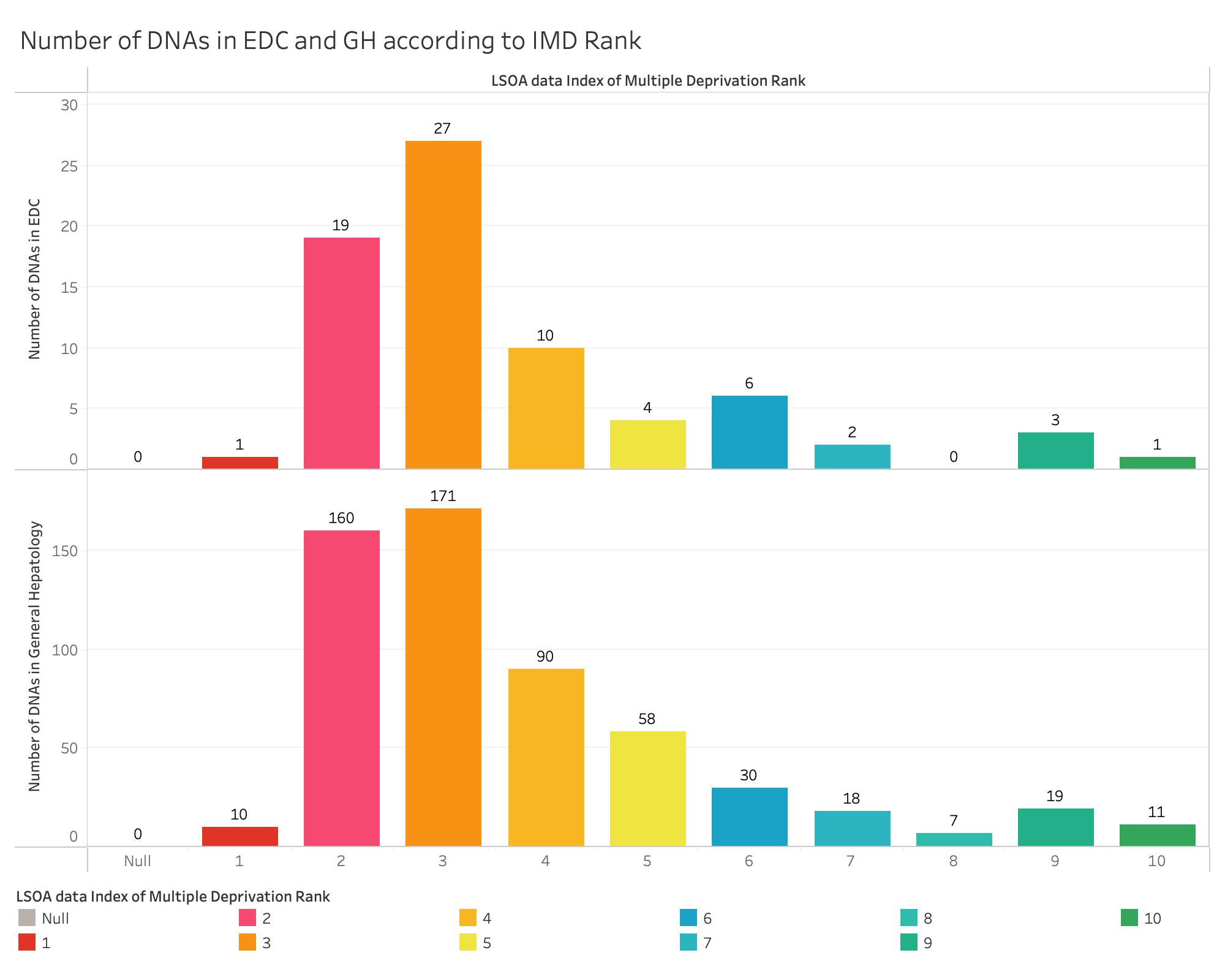
Supplementary Figure 2 – Top image: Number of DNAs at the Early diagnostic centre (EDC) stratified by Index of Multiple Deprivation (IMD) Rank


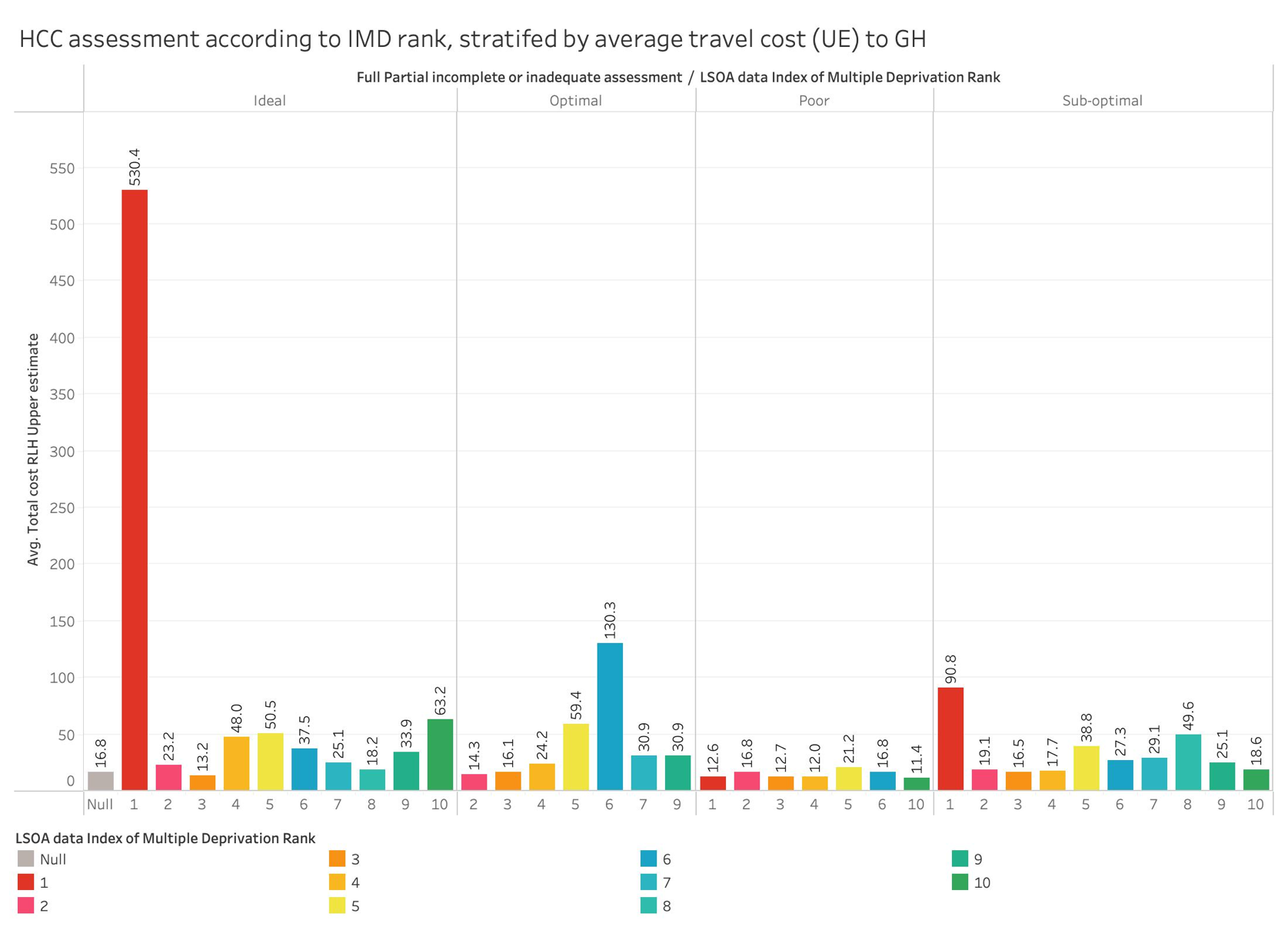


Supplementary Figure 3 - HCC surveillance assessment (Ideal/Optimal/Sub-optimal/poor) of all patients, according to Index of Multiple Deprivation (IMD) Rank stratified by average patient travel cost (upper estimate) to general hepatology (GH) clinic


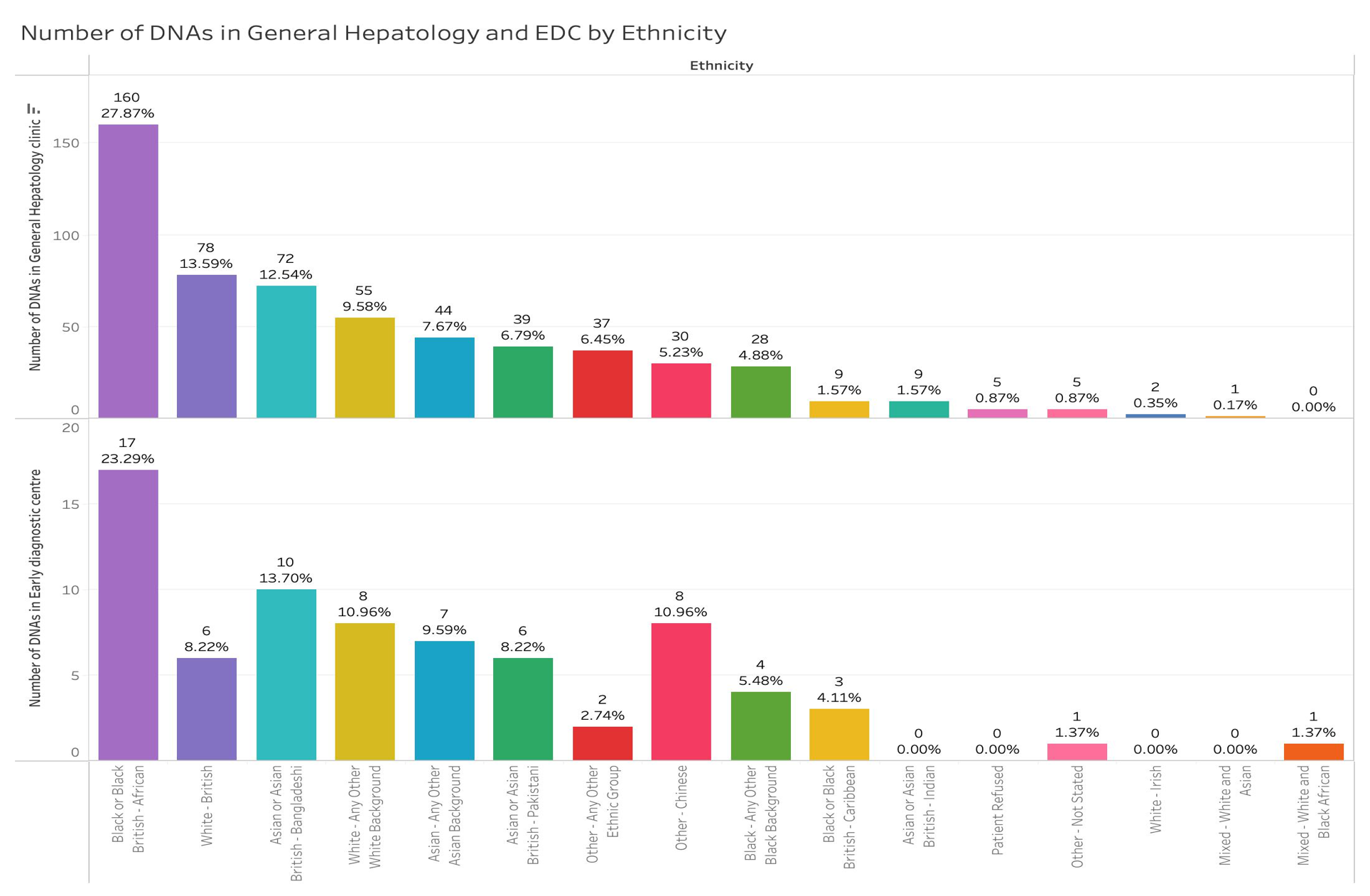


Supplementary Figure 4 - A: Number of DNAs in general hepatology (GH) categorised by ethnicity. B: Number of DNAs in the Early Diagnostic Centre (EDC) categorised by ethnicity


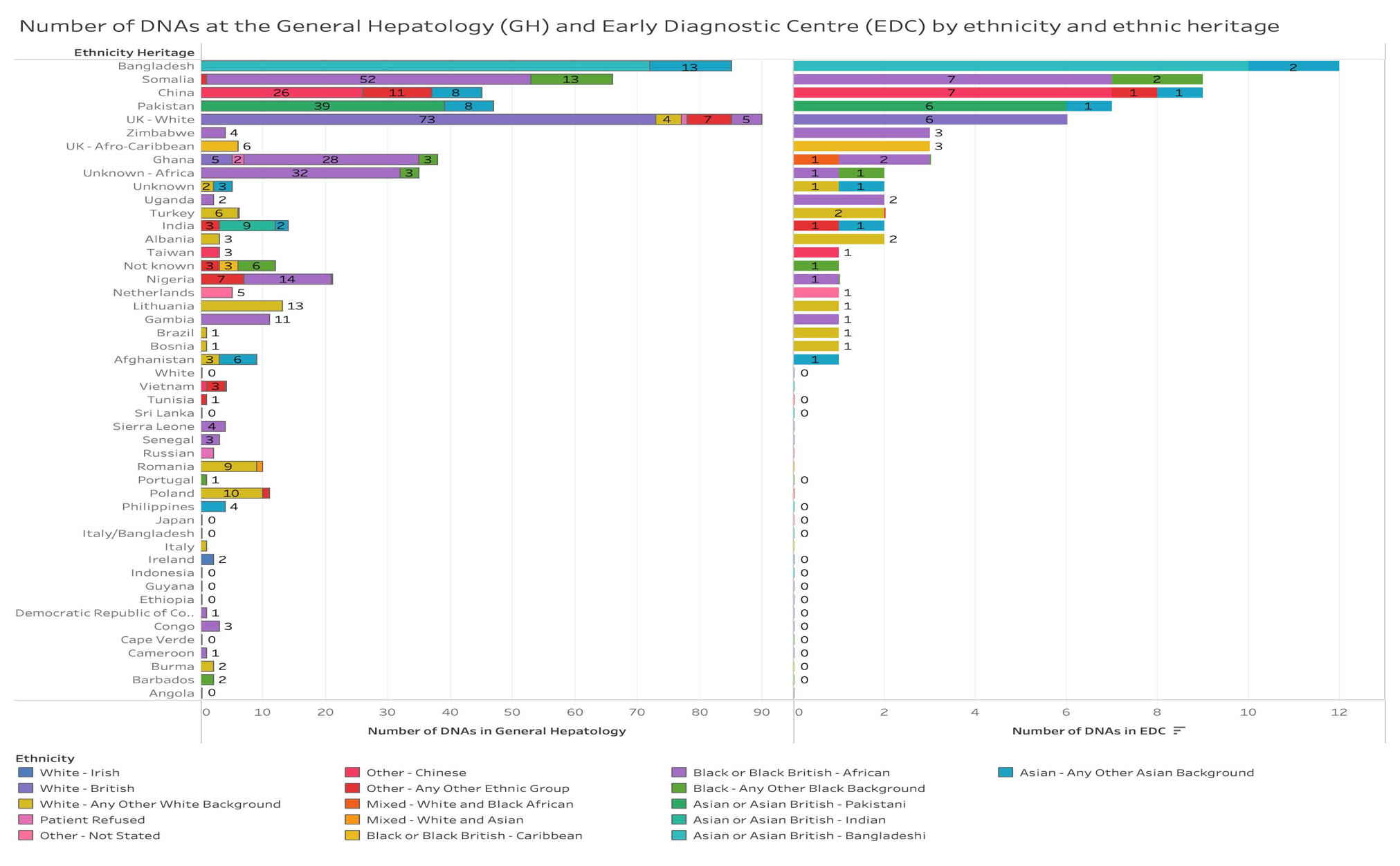


Supplementary Figure 5 - Number of DNAs at the General Hepatology (GH) and Early Diagnostic Centre (EDC) by ethnicity and ethnic heritage


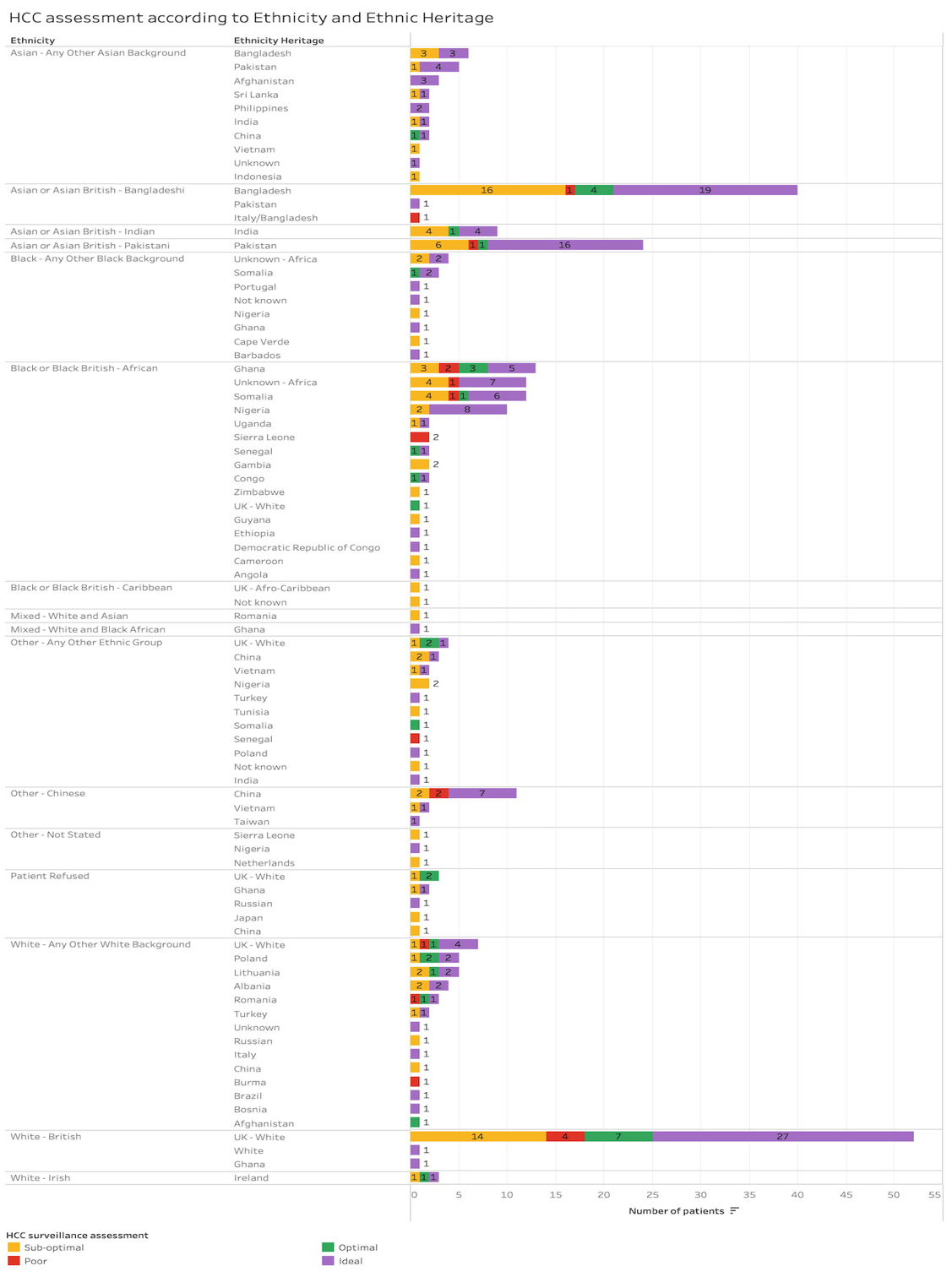


Supplementary Figure 6 - HCC surveillance assessment at the general hepatology according to Ethnic heritage of patient cohort with Ethnicity recorded via the NHS registration system and ethnic heritage determined from the patient notes


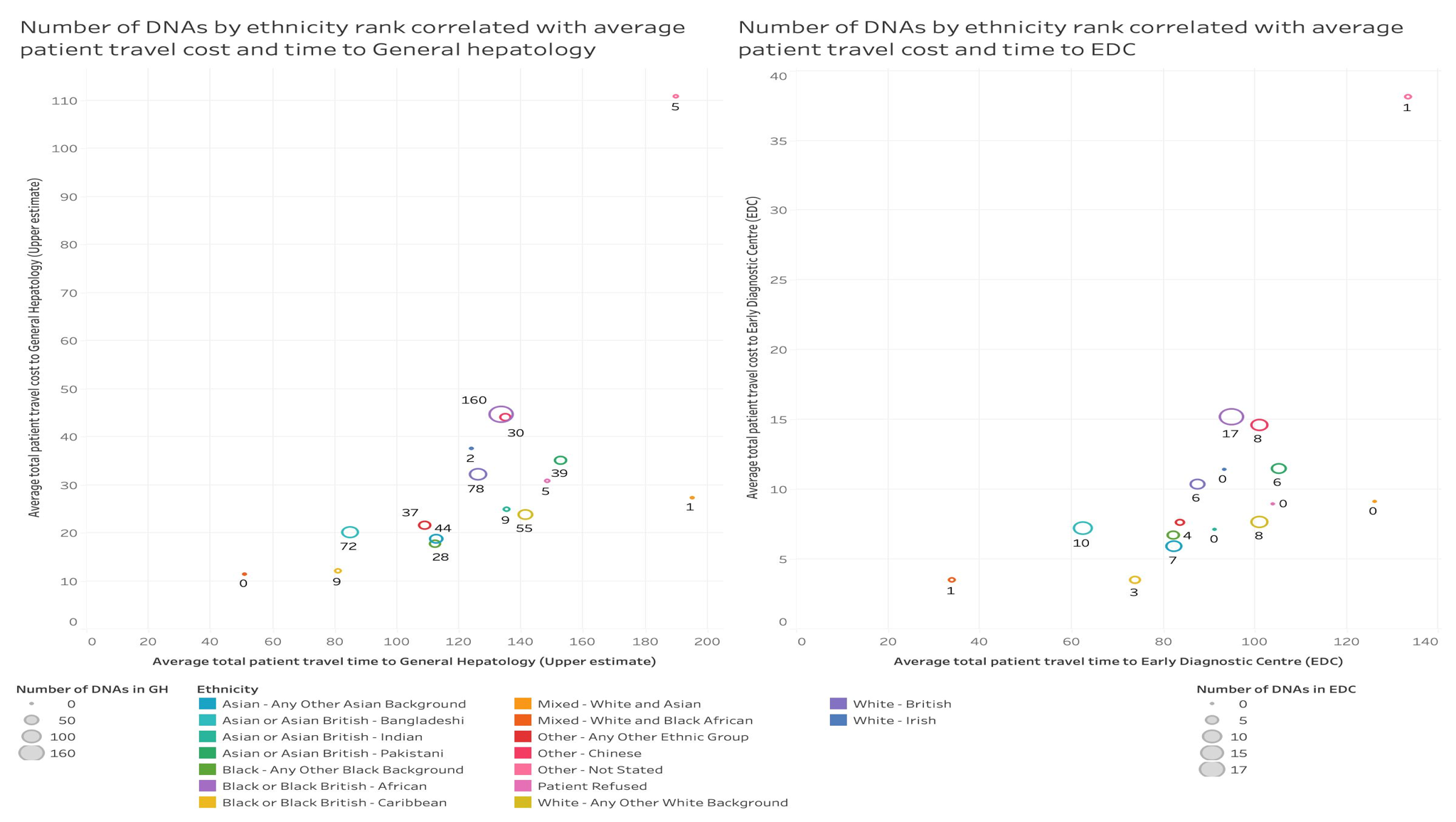


Supplementary Figure 7 – A - Number of DNAs stratified by ethnicity at the general hepatology clinic, correlated with average total patient cost and time (upper estimate) to the general hepatology clinic (GH); B - Number of DNAs stratified by ethnicity at Early Diagnostic Centre (EDC), correlated with total average patient time and cost to the EDC


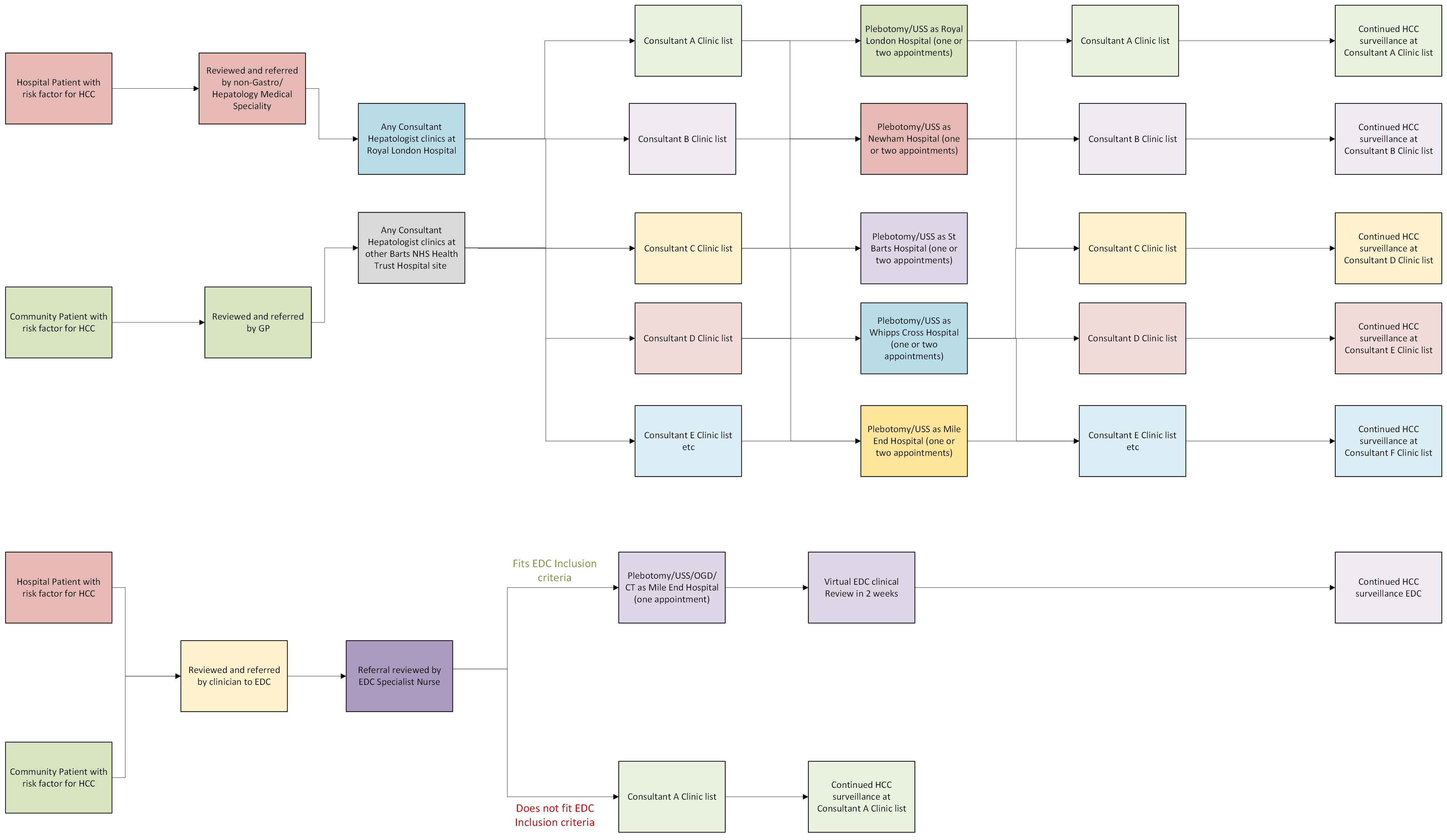


Supplementary Figure 8 - Graphical representation of the Early Diagnostic Centre (EDC) model compared to the previous model utilised in the Hepatology service
